# Environment-related health risks, knowledge and awareness among people in precarious milieus: secondary data analysis from the German “Environmental Awareness Study”

**DOI:** 10.3389/fpubh.2024.1369735

**Published:** 2024-10-09

**Authors:** Annabell Duda, Doreen Reifegerste

**Affiliations:** Bielefeld School of Public Health, Bielefeld University, Bielefeld, Germany

**Keywords:** environmental awareness, environmental knowledge, environmental behavior, environmental justice, precarious milieus, disadvantaged groups, environment-related health risks, secondary data analysis

## Abstract

**Introduction:**

Precarious milieus more frequently suffer from environmental risks and show lower environmental awareness and behavior than other milieus in the German population. This study investigates the factors that influence environmental awareness in precarious milieus and the roles of environmental knowledge and the perception of environment-related health burdens.

**Methods:**

A quantitative secondary data analysis of the German Environmental Awareness Study 2018 (*N* = 2017) was used to analyze the perception of environmental health burdens, environmental knowledge, and environmental awareness between precarious milieus (*n* = 190) and seven other milieus. One-way analysis of variance (ANOVA) and Bonferroni *post hoc* tests were used for this purpose. More in-depth analyses of the precarious milieus were carried out using multiple regression analyses.

**Results:**

There were significant differences in the perceptions of environmental health burdens affected by rail-traffic noise and neighborhood noise. Furthermore, environmental knowledge in precarious milieus was significantly lower than in five out of the seven other milieus (all *p* < 0.001) and was significantly associated with environmental cognition and gender. Precarious milieus had higher environmental affect than established milieus but less than that of critical-creative milieus and young idealists (all *p* < 0.001). Environmental cognition and environmental behavior were significantly associated with environmental affect. Environmental cognition was significantly higher in precarious milieus than in established milieus and among young pragmatists but was lower than in critical-creative milieus and among young idealists (all *p* < 0.001). Environmental affect, environmental knowledge, and gender were significantly associated with environmental cognition. In precarious milieus, environmental behavior was significantly lower than in traditional milieus and critical-creative milieus and among young idealists (all *p* < 0.001) and was significantly associated with environmental affect.

**Conclusion:**

The differences in the perception of environmental health burdens, environmental knowledge, and environmental awareness among precarious milieus indicate that there is a need for specific education and support structures for these population groups. Further research is needed to determine what other factors within the precarious milieus influence environmental knowledge and awareness, as well as the skills needed to understand environmental information, which are included in the framework of environmental (health) literacy.

## 1 Introduction

The connection between environmental risk factors and human health has been proven in many studies (1) and is referred to below as environmental health burden. It is known that air pollution and tobacco smoke are known to increase the risk of lung disease (2, 3), exposure to heavy metals (e.g., lead) or toxic chemicals (e.g., agricultural pesticides) is associated with neurodegenerative disorders such as Parkinson’s disease (4), and physical exposures such as extreme temperature and noise lead to cardiovascular mortality (5) and ischemic heart disease (6), respectively. However, environmental risks are not evenly distributed geographically or at population level, which is linked to environmental justice (7, 8). In particular, people with low socioeconomic status (precarious milieus) are more frequently affected by environmental risks, which reinforce existing social inequalities such as poverty or lack of education (9, 10). It is also known that people in precarious milieus have lower environmental awareness (11), which results in less pro-environmental behavior (12).

Depending on the definition, environmental awareness includes a combination of environmental affect (emotional involvement), environmental cognition (environmental attitudes) and environmental behavior (active action) (13). Furthermore, it either excludes or includes environmental knowledge (14), which is often understood as factual knowledge such as “Which action does not help to save energy costs in everyday life?” (15). Environmental knowledge is considered separately as it does not correlate strongly with the three sub-areas of environmental awareness (environmental affect, environmental cognition, and environmental behavior) in the context of the ‘Environmental Awareness Study’ (15), which has been surveying the environmental awareness and behavior of the German population for over 25 years (16).

The German population has shown high scores for environmental affect (7.2 out of 10 points) and environmental cognition (7.9 out of 10 points), but it has shown very low scores for environmental behavior (4.9 out of 10 points) and environmental knowledge (5.4 out of 10 points) (17). For example, the score for environmental behavior is below average among people in precarious milieus, while environmental affect and environmental cognition are average (information on environmental knowledge is not available) (17).

However, there is a lack of more detailed analyses on which factors within population groups influence the sub-components of environmental awareness. This is especially true for people in precarious milieus. In addition, there is also a lack of data on the role of environmental knowledge. This lack seems to especially troublesome when considering the high health burdens caused by environmental factors among people in precarious milieus. Therefore, the aim of this study was to investigate the environmental awareness and knowledge of people in precarious milieus, as well as their perception of environment-related health burdens in order to analyze the issues of both environment and health together. This is based on the following research questions (RQs):

RQ1: How does the perception of *environmental health burdens* differ between people from precarious milieus and other milieus?

RQ2: How does *environmental knowledge* differ between people from precarious milieus and other milieus?

RQ3: Which determinants influence *environmental knowledge* within the precarious milieus?

RQ4: How does *environmental awareness* differ between people from precarious milieus and other milieus?

RQ5: Which determinants influence *environmental awareness* within the precarious milieus?

## 2 Materials and methods

### 2.1 Data source and study sample

This study involved a secondary analysis of data (18) from the 2018 wave of the representative German Environmental Awareness Study on mobility, agriculture, and energy transition (17). The procedure of this analysis was based on the guidelines and recommendations (“Good Practice Secondary Data Analysis”) of the Working Group on the Collection and Use of Secondary Data (AGENS) of the German Society for Social Medicine and Prevention (DGSMP) and the German Society for Epidemiology (DGEpi) (19). The German Federal Ministry for the Environment, Nature Conservation, Nuclear Safety, and Consumer Protection (BMUV) and the German Federal Environmental Agency (UBA) has regularly commissioned the representative survey since 1996. The respective publications provide details on participant recruitment, procedures and reporting of full outcomes (20). The 2018 Environmental Awareness Study consisted of a main study that was conducted as an online survey in two waves with more than 2,000 respondents each, as well as a qualitative preliminary study (17). The present study used only the data from the wave in which environment-related health burdens were collected. Of the *N* = 2017 respondents, 50% (*n* = 1,008) reported being female, 49.9% (*n* = 996) reported being male, and 0.3% (*n* = 7) reported being Inter* or Trans*. The average age was 49.55 years (range: 14 to 91 years).

To examine status groups, the 2018 Environmental Awareness Study used the social milieu model from the Institute for Socio-cultural Research (sociodimensions) (21). It distinguishes five milieus and three youth segments based on different everyday cultures, lifeworlds, and social situations. Within a social milieu, similar values, principles of lifestyle, and similar mentalities dominate. As a result, the influence of the living environment is taken into account when considering individual environmental awareness. By looking at social milieus, the different everyday cultures, lifeworlds and values are included, so that this type of consideration goes beyond a pure age and gender consideration.

The largest milieu is the *bourgeois mainstream*, representing 26% (*n* = 488) of the study population. People who belong to this milieu usually have a medium social situation, medium education, and medium income. The most important thing for these people is their private and family life. The *established milieus* (14%, *n* = 328) are in the same generational situation as the bourgeois mainstream, but they have a higher social situation. People in this milieu usually have a medium to high level of education and high to very high income. Men are more strongly represented in this milieu than women. These people value a high standard of living and are therefore performance and success-oriented. People over 70 years old are increasingly found in traditional milieus. For demographic reasons, women are overrepresented in this milieu. The social situation varies greatly and ranges from low to high. The common factor is that *traditional milieus* (14%, *n* = 316) form a basic attitude. The focus of this milieu is on security, stability, and thriftiness, which is associated with a willingness to make sacrifices. People from *precarious milieus* (13%, *n* = 190) represent the population with the lowest social status. They usually have a low level of education and very low to low income. Single mothers are overrepresented in this milieu. The age groups over 40 years old are overrepresented, purchasing power and participation in social life is severely limited. The focus is on coping with everyday routines, providing for the family, and keeping a job.

In contrast, people from *critical-creative milieus* (13%, *n* = 319) have the highest social position. The age spectrum is very broad and includes 30 to 70 year-olds. They have a medium to high level of education and medium to high income. Women are clearly overrepresented. People in this milieu are cosmopolitan and tolerant, and independence and self-realization are important to them. The three young segments are the young idealists, young pragmatists, and young detached. These segments differ in that *young idealists* (5%, *n* = 113) want to participate in socio-ecological change and show a high level of commitment. *Young pragmatists* (9%, *n* = 171) are more concerned with professional success and a good standard of living. The *young detached* (5%, *n* = 95) tend to have a low social position and perceive everyday tasks as a challenge (21).

The most recent data from the 2020 Environmental Awareness Study could not be used because it analyzed types of environmental awareness (12) not milieus. Unfortunately, replication of the milieus was not possible due to a lack of comparable indicator variables.

### 2.2 Study variables

#### 2.2.1 Milieu operationalization

The operationalization to milieus was included as a variable in the used dataset. Operationalization is based on a total score resulting from the highest level of education, monthly income, and occupational group. For example, the variable income is coded as follows: less than EUR 1,000 (1 score point), 1,000 to less than EUR 1,500 (2 score points), 1,500 to less than EUR 2,000 (3 score points), 2,000 to less than EUR 3,000 (4 score points), 3,000 to under EUR 4,000 (5 score points) and more than EUR 4,000 (6 score points). The same principle is used to code education and occupational group. The education category ranges from 1 score point for no school-leaving qualification to 5 score points for a university degree. The occupational group categories included 1 score point for ordinary employees and civil servants in elementary service, 2 score points for skilled employees and civil servants in mid-level service, and 3 score points for qualified or managerial employees and civil servants in upper or higher service and liberal professions.

These three scales are used to form a sum score (minimum = 3, maximum = 14), which is divided into four quartiles: lower social class (3–5 score points), lower middle class (6–8 score points), upper middle class (9–11 score points) and upper social class (12–14 score points). For this reason, there is no direct operationalization of concepts such as educational qualifications to a social situation, but only aggregated values. This means that a medium social situation, medium education, and medium income cannot be described based on specific qualifications or income values. More details can be found in the methodology report by Socio-cultural Research (21).

There were also 8 items to determine the social milieus (e.g., “I have enough to do with my own problems, I cannot take care of others”). The answers were based on a 5-point scale ranging from “totally agree” to “not know”.

#### 2.2.2 Environment-related health burdens

The environmental health burdens are based on self-report. General health burden connected with environmental problems and the health burden connected with various noise sources were surveyed. The general health burden was measured using a 5-point scale (ranging from “very strongly” to “not at all”). Questions on noise exposure asked about how much the respondent had been disturbed or annoyed in the last 12 months by rail-traffic noise, road traffic noise, air traffic noise, industrial noise/commercial noise, and noise from neighbors. The answers were based on a 6-point scale ranging from “extremely disturbed” to “not disturbed at all”.

#### 2.2.3 Environmental knowledge

Environmental knowledge is measured objectively as falsifiable factual knowledge. The degree of informedness and the conviction of one’s own knowledge are not recorded. Based on the work of Geiger et al. (15), which includes 35 items to capture environmental knowledge, 10 items were selected for the 2018 Environmental Awareness Study based on statistical selection procedures. This short scale has adequate internal consistency (Cronbach’s *α* ≧ 0.7) (15). For each knowledge question, there were four possible answers, of which only one was correct. Knowledge questions in the 2018 Environmental Awareness Study covered the topics: renewable energies, household energy consumption, the greenhouse effect, short-distance staff transport, soil fertility, impervious surfaces in Europe, groundwater pollution in the EU, air pollution, sustainability, and the Paris Climate Convention.

#### 2.2.4 Environmental awareness

For the 2018 Environmental Awareness Study, an existing environmental awareness scale based on the pressure-state response model was revised to include 23 items: seven items on environmental affect, eight items on environmental cognition, and eight items on environmental behavior. The affective and cognitive items are measured using a 4-point agreement scale ranging from “totally agree” to “completely disagree,” and the behavioral scale involves a 6-point frequency scale [from 6 (always) to 1 (never)] and three dichotomous items (yes or no). The internal consistency of the environmental awareness scale was adequate (Cronbach’s *α* = 0.71).

### 2.3 Statistical analysis

All analyses were performed using SPSS [version 28.0.1.0 (142)] (22). First, descriptive analyses were conducted to determine the characteristics of the precarious milieus. In addition, further descriptive analyses were conducted to calculate frequencies and means for all variables of interest for the precarious milieus and the seven other milieus. Second, one-way analysis of variance (ANOVA) and Bonferroni *post hoc* tests were calculated to examine differences between milieus. Third, in-depth analyses of the correlations between environmental knowledge and the three sub-components of environmental awareness (Pearson correlations) were carried out on the precarious milieus. Based on the significant results of the Pearson correlation, multiple regression analyses were performed as a final step.

## 3 Results

### 3.1 Differences in the perception of environmental health burdens

Regarding RQ1, the ANOVA showed a significant difference in the general perception of environmental health burdens between the milieus [*F*(7,1915) = 5.841, *p* < 0.001, *n* = 1923]. The Bonferroni *post hoc* test (see Table 1) showed significant differences between the precarious milieus and the established milieus (*p* < 0.001). People in precarious milieus (*M* = 2.48, *SD* = 0.69) more frequently stated that they feel stressed by the environmental health burdens than those in the established milieus (*M* = 2.20, *SD* = 0.70).

**Table 1:** Bonferroni *post hoc* tests of general perception of environmental health burdens by milieus.

(I) Milieu	(J) Milieus	Mean difference (I-J)	Std. error	Sign.	95% CI	
					LL	UL
Precarious milieus (*n* = 190)	Traditional milieus (*n* = 316)	−0.150	0.066	0.663	−0.36	0.06
	Established milieus (*n* = 328)	−0.278	0.064	**<0.001**	−0.48	−0.08
	Bourgeois mainstream (*n* = 488)	−0.160	0.063	0.306	−0.36	0.04
	Critical-creative milieus (*n* = 319)	−0.003	0.068	1.000	−0.22	0.21
	Young idealists (*n* = 113)	0.035	0.087	1.000	−0.24	0.31
	Young pragmatists (*n* = 171)	−0.183	0.074	0.380	−0.41	0.05
	Young detached (*n* = 92)	−0.171	0.087	1.000	−0.44	0.10

Significant differences (*p* ≤ 0.05) are highlighted in bold.

A more differentiated view of the environmental health burdens of noise showed that there are also differences between the milieus in personal perception of the extent to which rail-traffic noise [*F*(7,1997) = 4.789, *p* < 0.001, *n* = 2005], road traffic noise [*F*(7,2010) = 6.401, *p* < 0.001, *n* = 2018], air traffic noise [*F*(7,2000) = 5.798, *p* < 0.001, *n* = 2008], industrial and commercial noise [*F*(7,1990) = 5.152, *p* < 0.001, *n* = 1998], and neighborhood noise [*F*(7,2005) = 8.193, *p* < 0.001, *n* = 2013] are perceived as disturbing or annoying. The results of the Bonferroni *post hoc* test can be found in Tables 2, 3. Significant differences between precarious milieus and other milieus could be identified for only two sources of noise. First, rail-traffic noise was perceived as less disturbing or annoying by those in precarious milieus (*M* = 1.55, *SD* = 0.93) than by the young detached (*M* = 1.98, *SD* = 1.23, *p* = 0.009). Second, those in precarious milieus (*M* = 2.29, *SD* = 1.26) perceived neighborhood noise as more disturbing or annoying than those in traditional milieus (*M* = 1.75, *SD* = 1.02, *p* < 0.001). No significant differences were found between the precarious milieus and the other milieus with regard to exposure to road traffic noise, air traffic noise, and industrial and commercial noise. As no patterns could be identified in the analysis of the perception of environmental health burdens, this variable was excluded from the regression analyses for RQ3 and RQ5.

**Table 2:** Bonferroni *post hoc* tests of rail-traffic noise by milieus.

(I) Milieu	(J) Milieus	Mean difference (I-J)	Std. error	Sign.	95% CI	
					LL	UL
Precarious milieus (*n* = 190)	Traditional milieus (*n* = 316)	−0.099	0.088	1.000	−0.37	0.18
	Established milieus (*n* = 328)	0.018	0.086	1.000	−0.25	0.29
	Bourgeois mainstream (*n* = 488)	0.023	0.084	1.000	−0.24	0.29
	Critical-creative milieus (*n* = 319)	0.161	0.091	1.000	−0.12	0.45
	Young idealists (*n* = 113)	0.227	0.118	1.000	−0.14	0.60
	Young pragmatists (*n* = 171)	0.080	0.100	1.000	−0.23	0.39
	Young detached (*n* = 92)	0.429	0.119	**0.009**	0.06	0.80

Significant differences (*p* ≤ 0.05) are highlighted in bold.

**Table 3:** Bonferroni *post hoc* tests of neighborhood noise by milieus.

(I) Milieu	(J) Milieus	Mean difference (I-J)	Std. error	Sign.	95% CI	
					LL	UL
Precarious milieus (*n* = 190)	Traditional milieus (*n* = 316)	−0.536	0.110	**<0.001**	−0.88	−0.19
	Established milieus (*n* = 328)	−0.236	0.108	0.825	−0.57	0.10
	Bourgeois mainstream (*n* = 488)	−0.062	0.105	1.000	−0.39	0.27
	Critical-creative milieus (*n* = 319)	−0.072	0.114	1.000	−0.43	0.28
	Young idealists (*n* = 113)	0.126	0.148	1.000	−0.34	0.59
	Young pragmatists (*n* = 171)	−0.006	0.125	1.000	−0.40	0.38
	Young detached (*n* = 92)	0.079	0.149	1.000	−0.39	0.54

Significant differences (*p* ≤ 0.05) are highlighted in bold.

### 3.2 Differences and determinants of environmental knowledge

With regard to RQ2, the ANOVA showed a significant difference in environmental knowledge between the milieus [*F*(7,2009) = 14,752, *p* < 0.001, *n* = 2017]. The Bonferroni *post hoc* test (see Table 4) showed significant differences (all *p* < 0.001) between the precarious milieus (*M* = 4.84, *SD* = 1.93) and traditional milieus (*M* = 5.52, *SD* = 1.67), established milieus (*M* = 5.89, *SD* = 1.57), the bourgeois mainstream (*M* = 5.43, *SD* = 1.65), and critical-creative milieus (*M* = 6.08, *SD* = 1.55), as well as between precarious milieus and young idealists (*M* = 5.81, *SD* = 1.50). Compared to these other milieus, those in precarious milieus were less likely to answer the knowledge questions correctly. There were no significant differences between the precarious milieus and the young pragmatists and young detached.

**Table 4:** Bonferroni *post hoc* tests of environmental knowledge by milieus.

(I) Milieu	(J) Milieus	Mean difference (I-J)	Std. error	Sign.	95% CI	
					LL	UL
Precarious milieus (*n* = 190)	Traditional milieus (*n* = 316)	−0.685	0.151	**<0.001**	−1.16	−0.21
	Established milieus (*n* = 328)	−1.050	0.150	**<0.001**	−1.52	−0.58
	Bourgeois mainstream (*n* = 488)	−0.596	0.141	**<0.001**	−1.04.	−0.15
	Critical-creative milieus (*n* = 319)	−1.238	0.151	**<0.001**	−1.71	−0.77
	Young idealists (*n* = 113)	−0.968	0.196	**<0.001**	−1.58	−0.36
	Young pragmatists (*n* = 171)	−0.298	0.174	1.000	−0.84	0.25
	Young detached (*n* = 92)	−0.304	0.210	1.000	−0.96	0.35

Significant differences (*p* ≤ 0.05) are highlighted in bold.

Regarding RQ3, the multiple regression (see Table 5) showed that the predictors environmental cognition (*p* = 0.008) and gender (*p* < 0.001) were significantly associated with the criterion environmental knowledge within the precarious milieus [*F*(8,167) = 4.197, *p* < 0.001, *n* = 190]. Educational qualification, age, environmental affect, environmental behavior, and monthly income were not significantly related.

**Table 5:** Coefficient table of the multiple regression analysis of the environmental knowledge and the socio-demographic variables within the precarious milieus.

Effect	Beta	SE	95% CI		Sign.
			LL	UL	
Environmental affect	−0.056	0.119	−0.295	0.175	0.617
Environmental cognition	0.283	0.136	0.095	0.632	**0.008**
Environmental behavior	0.115	0.097	−0.066	0.318	0.197
Male	0.366	0.285	0.819	1.943	**<0.001**
Inter*/Trans*	0.093	1.326	−1.014	4.223	0.228
Educational qualification	0.028	0.820	−1.308	1.928	0.706
Monthly income	0.007	0.108	−0.203	0.225	0.918
Age	0.111	0.106	−0.050	0.370	0.135

Environmental knowledge (min = 0, max = 10), Influencing variables: Dummy coding Gender 0 = not male and 1 = male, 0 = not Inter*/Trans*, 1 = Inter*/Trans*, Educational qualification (low, medium, high), Monthly income (under 1,000€, 1,000€ up to 2000€, 2000€ up to 3,000€, 3,000€ up to 4,000€, 4,000€ and more), Age (14–19 years, 20–29 years, 30–39 years, 40–49 years, 50–59 years, 60–69 years, 70 years and older), *R*^2^ = 0.167, Significant differences (*p* ≤ 0.05) are highlighted in bold.

### 3.3 Differences and determinants of environmental awareness

The ANOVA showed that the milieus had a significant difference in environmental affect [*F*(7,2001) = 73.823, *p* < 0.001, *n* = 2009], environmental cognition [*F*(7,2000) = 82.145, *p* < 0.001, *n* = 2008], and environmental behavior [*F*(7,2009) = 67.063, *p* < 0.001, *n* = 2017].

The differences and determinants of the sub-components of environmental awareness (environmental affect, environmental cognition and environmental behavior) are discussed in more detail below with regard to RQ4 and RQ5.

#### 3.3.1 Differences and determinants of environmental affect

It can be seen that precarious milieus differ significantly from established milieus (*p* < 0.001), critical-creative milieus (*p* < 0.001) and young idealists (*p* < 0.001) in terms of their environmental affect. While the precarious milieus (*M* = 6.98, *SD* = 1.72) have a higher environmental affect than the established milieus (*M* = 6.28, *SD* = 2.03), this value is lower than that of critical-creative milieus (*M* = 8.73, *SD* = 1.16) and young idealists (*M* = 8.79, *SD* = 0.99). The corresponding Bonferroni *post hoc* results are presented in Table 6.

**Table 6:** Bonferroni *post hoc* tests of environmental affect by milieus.

(I) Milieu	(J) Milieus	Mean Difference (I-J)	Std. Error	Sign.	95% CI	
					LL	UL
Precarious milieus (*n* = 186)	Traditional milieus (*n* = 316)	−0.26803	0.15901	1.000	−0.7654	0.2293
	Established milieus (*n* = 328)	0.70023	0.15793	**<0.001**	0.2062	1.1942
	Bourgeois mainstream (*n* = 486)	0.26685	0.14835	1.000	−0.1972	0.7309
	Critical-creative milieus (*n* = 319)	−1.75450	0.15873	**<0.001**	−2.2510	−1.2580
	Young idealists (*n* = 113)	−1.81057	0.20522	**<0.001**	−2.4525	−1.1687
	Young pragmatists (*n* = 171)	0.50621	0.18229	0.155	−0.0640	1.0764
	Young detached (*n* = 90)	−2.4876	0.22093	1.000	−0.9398	0.4423

Significant differences (*p* ≤ 0.05) are highlighted in bold.

The multiple regression (see Table 7) showed that the predictors environmental cognition (*p* < 0.001) and environmental behavior (*p* < 0.001) were significantly associated with the criterion environmental affect within the precarious milieus [*F*(8,167) = 31.905, *p* < 0.001, *n* = 186]. Educational qualification, age, environmental knowledge, gender, and monthly income were not significantly related.

**Table 7:** Coefficient table of the multiple regression analysis of the environmental affect and the socio-demographic variables within the precarious milieus.

Effect	Beta	SE	95% CI		Sign.
			LL	UL	
Environmental cognition	0.589	0.072	0.570	0.853	**<0.001**
Environmental behavior	0.308	0.059	0.203	0.434	**<0.001**
Environmental knowledge	−0.027	0.050	−0.124	0.074	0.617
Male	0.006	0.198	−0.368	0.412	0.911
Inter*/Trans*	−0.037	0.864	−2.300	1.112	0.493
Educational qualification	−0.035	0.532	−1.411	0.690	0.499
Monthly income	−0.012	0.070	−0.156	0.123	0.815
Age	−0.099	0.069	−0.270	0.002	0.053

Environmental affect (min = 0, max = 10), Influencing variables: Dummy coding Gender 0 = not male and 1 = male, 0, 0 = not Inter*/Trans*, 1 = Inter*/Trans*, Educational qualification (low, medium, high), Monthly income (under 1,000€, 1,000€ up to 2000€, 2000€ up to 3,000€, 3,000€ up to 4,000€, 4,000€ and more), Age (14–19 years, 20–29 years, 30–39 years, 40–49 years, 50–59 years, 60–69 years, 70 years and older), Environmental knowledge (min = 0, max = 10), Environmental cognition (min = 0, max = 10), Environmental behavior (min = 0, max = 10), *R*^2^ = 0.604, Significant differences (*p* ≤ 0.05) are highlighted in bold.

#### 3.3.2 Differences and determinants of environmental cognition

In terms of environmental cognition, precarious milieus differed significantly from established milieus (*p* < 0.001), critical-creative milieus (*p* < 0.001), young idealists (*p* < 0.001), and young pragmatists (*p* < 0.001). Compared to the established milieus (*M* = 7.16, *SD* = 1.37) and the young pragmatists (*M* = 6.83, *SD* = 1.63), the precarious milieus (*M* = 7.77, *SD* = 1.41) had higher environmental cognition. In contrast to the critical-creative milieus (*M* = 9.06, *SD* = 0.79) and the young idealists (*M* = 8.78, *SD* = 0.96), environmental cognition was lower in the precarious milieus. The corresponding Bonferroni *post hoc* results are presented in Table 8.

**Table 8:** Bonferroni *post hoc* tests of environmental cognition by milieus.

(I) Milieu	(J) Milieus	Mean difference (I-J)	Std. error	Sign.	95% CI	
					LL	UL
Precarious milieus (*n* = 185)	Traditional milieus (*n* = 316)	−0.24494	0.11660	1.000	−0.6097	0.1198
	Established milieus (*n* = 328)	0.60468	0.11581	**<0.001**	0.2424	0.9669
	Bourgeois mainstream (*n* = 486)	0.02885	0.10881	1.000	−0.3115	0.3692
	Critical-creative milieus (*n* = 319)	−1.29532	0.11640	**<0.001**	−1.6594	−0.9312
	Young idealists (*n* = 113)	−1.01286	0.15038	**<0.001**	−1.4832	−0.5425
	Young pragmatists (*n* = 171)	0.93340	0.13361	**<0.001**	0.5155	1.3513
	Young detached (*n* = 90)	−0.00854	0.16187	1.000	−0.5149	0.4978

Significant differences (*p* ≤ 0.05) are highlighted in bold.

The multiple regression (see Table 9) showed that the predictors environmental affect (*p* < 0.001), environmental knowledge (*p* = 0.008), and gender (*p* = 0.005) were significantly associated with the criterion environmental cognition within the precarious milieus, [*F*(8,167) = 28.354, *p* < 0.001, *n* = 185]. Educational qualification, age, environmental behavior, and monthly income were not significantly related.

**Table 9:** Coefficient table of the multiple regression analysis of the environmental cognition and the socio-demographic variables within the precarious milieus.

Effect	Beta	SE	95% CI		Sign.
			LL	UL	
Environmental affect	0.632	0.053	0.419	0.627	**<0.001**
Environmental behavior	0.056	0.054	−0.059	0.155	0.380
Environmental knowledge	0.144	0.042	0.029	0.196	**0.008**
Male	−0.160	0.165	−0.797	−0.144	**0.005**
Inter*/Trans*	−0.083	0.737	−2.569	0.341	0.133
Educational qualification	0.013	0.457	−0.792	1.012	0.810
Monthly income	0.026	0.060	−0.088	0.150	0.606
Age	0.060	0.059	−0.050	0.184	0.261

Environmental cognition (min = 0, max = 10), Influencing variables: Dummy coding Gender 0 = not male and 1 = male, 0 = not Inter*/Trans*, 1 = Inter*/Trans*, Educational qualification (low, medium, high), Monthly income (under 1,000€, 1,000€ up to 2000€, 2000€ up to 3,000€, 3,000€ up to 4,000€, 4,000€ and more), Age (14–19 years, 20–29 years, 30–39 years, 40–49 years, 50–59 years, 60–69 years, 70 years and older), Environmental knowledge (min = 0, max = 10), Environmental affect (min = 0, max = 10), Environmental behavior (min = 0, max = 10), *R*^2^ = 0.576, Significant differences (*p* ≤ 0.05) are highlighted in bold.

#### 3.3.3 Differences and determinants of environmental behavior

The environmental behavior of people in precarious milieus differs significantly from that of traditional milieus (*p* < 0.001), critical-creative milieus (*p* < 0.001), and young idealists (*p* < 0.001). Precarious milieus (*M* = 4.09, *SD* = 1.71) showed less environmental behavior than the traditional milieus (*M* = 4.96, *SD* = 1.55), critical-creative milieus (*M* = 6.10, *SD* = 1.67), and young idealists (*M* = 5.70, *SD* = 1.54). The corresponding Bonferroni *post hoc* results are presented in Table 10.

**Table 10:** Bonferroni *post hoc* tests of environmental behavior by milieus.

(I) Milieu	(J) Milieus	Mean difference (I-J)	Std. error	Sign.	95% CI	
					LL	UL
Precarious milieus (*n* = 190)	Traditional milieus (*n* = 316)	−0.86951	0.14351	**<0.001**	−1.3184	−0.4206
	Established milieus (*n* = 328)	−0.32815	0.14252	0.599	−0.7739	0.1176
	Bourgeois mainstream (*n* = 488)	−0.19767	0.13367	1.000	−0.6158	0.2204
	Critical-creative milieus (*n* = 319)	−2.00931	0.14325	**<0.001**	−2.4574	−1.5612
	Young idealists (*n* = 113)	−1.60405	0.18570	**<0.001**	−2.1849	−1.0232
	Young pragmatists (*n* = 171)	0.33029	0.16478	1.000	−0.1851	0.8457
	Young detached (*n* = 92)	0.18851	0.19855	1.000	−0.4325	0.8096

Significant differences (*p* ≤ 0.05) are highlighted in bold.

The multiple regression (see Table 11) showed that only the predictor environmental affect (*p* < 0.001) was significantly associated with the criterion environmental behavior within the precarious milieus [*F*(8,167) = 12.270, *p* < 0.001, *n* = 190]. Educational qualification, age, gender, environmental knowledge, environmental cognition, and monthly income were not significantly related.

**Table 11:** Coefficient table of the multiple regression analysis of the environmental behavior and the socio-demographic variables within the precarious milieus.

Effect	Beta	SE	95% CI		Sign.
			LL	UL	
Environmental affect	0.490	0.087	0.302	0.645	**<0.001**
Environmental cognition	0.083	0.110	−0.120	0.313	0.380
Environmental knowledge	0.087	0.061	−0.041	0.199	0.197
Male	−0.087	0.240	−0.773	0.173	0.212
Inter*/Trans*	0.038	1.054	−1.481	2.680	0.570
Educational qualification	−0.066	0.647	−1.934	0.622	0.312
Monthly income	−0.025	0.086	−0.204	0.135	0.685
Age	0.080	0.084	−0.062	0.272	0.215

Environmental behavior (min = 0, max = 10), Influencing variables: Dummy coding Gender 0 = not male and 1 = male, 0 = not Inter*/Trans*, 1 = Inter*/Trans*, Educational qualification (low, medium, high), Monthly income (under 1,000€, 1,000€ up to 2000€, 2000€ up to 3,000€, 3,000€ up to 4,000€, 4,000€ and more), Age (14–19 years, 20–29 years, 30–39 years, 40–49 years, 50–59 years, 60–69 years, 70 years and older), Environmental knowledge (min = 0, max = 10), Environmental affect (min = 0, max = 10), Environmental cognition (min = 0, max = 10), *R*^2^ = 0.370, Significant differences (*p* ≤ 0.05) are highlighted in bold.

## 4 Discussion

In view of existing environmental changes and their consequences for health, studies on the topic of environment and health are highly relevant, especially from an environmental justice perspective. However, there is a lack of in-depth analyses of environmental knowledge and the sub-components of environmental awareness within population groups. This research gap exists especially with regard to people in precarious milieus, which we aimed to address with this secondary data analysis. For this reason, this study investigated the subjective perception of environment-related health burdens in general and in relation to noise, environmental knowledge, and the sub-components of environmental awareness (environmental affect, environmental cognition and environmental behavior) of precarious milieus and seven other milieus.

In general, people in precarious milieus felt significantly more affected by environmental health burdens than those in established milieus (*p* < 0.001). Considering the perception of environmental health burdens caused by noise, it can be seen that people in precarious milieus felt significantly less affected by rail-traffic noise than young detached (*p* = 0.009) but significantly more than those in traditional milieus with regard to neighborhood noise (*p* < 0.001). In addition, precarious milieus had significantly lower environmental knowledge than those representing traditional milieus, established milieus, the bourgeois mainstream, critical-creative milieus, and young idealists (all *p* < 0.001). The multiple regression analysis showed that environmental cognition (*p* = 0.008) and gender (*p* < 0.001) were significantly associated with the criterion of environmental knowledge within the precarious milieus. Significant differences were found in all three sub-components of environmental awareness. Precarious milieus had higher environmental affect than established milieus but lower than that of critical-creative milieus and young idealists (all *p* < 0.001). Based on the multiple regression analysis, environmental cognition (*p* < 0.001), and environmental behavior (*p* < 0.001) were significantly associated with environmental affect within the precarious milieus. Environmental cognition was significantly higher in precarious milieus than in established milieus and among young pragmatists but lower than in critical-creative milieus and among young idealists (all *p* < 0.001). The multiple regression analysis showed that environmental affect (*p* < 0.001), environmental knowledge (*p* = 0.008), and gender (*p* = 0.005) were significantly associated with environmental cognition within the precarious milieus. Furthermore, significant differences in environmental behavior were also found. Precarious milieus had significantly lower environmental behavior than traditional milieus, critical-creative milieus, and young idealists (all *p* < 0.001). According to the multiple regression analysis, environmental affect (*p* < 0.001) was significantly associated with environmental behavior within the precarious milieus.

The literature shows that people from precarious milieus are more often affected by environmental health risks (9). Our results reveal statistical differences in only the perception of environment-related health burdens in general between precarious milieus and established milieus (*p* < 0.001). These two milieus were very similar in terms of socio-historical characteristics, such as generational factors, which was why these milieus were good for comparison with each other. They differed mainly in that precarious milieus had a low social situation, while established milieus had a high social situation (21), which could provide a possible explanation. Groups with lower socioeconomic position have higher exposure to environmental noise (23). Surprisingly, however, no significant results were found between precarious milieus and established milieus with regard to perceived noise. This suggests that other environmental factors were perceived as a burden on health in established milieus.

The perception of rail-traffic noise differed significantly only between precarious milieus and the young detached (*p* = 0.009), and that of neighborhood noise differed only between precarious milieus and traditional milieus (*p* < 0.001). Precarious milieus and the young detached have similar social situations in that the social situation of the young detached is more likely to be classified between a low and a medium. The two milieus differ primarily in terms of age as the young detached tend to be between 14 and 30 years old, while the number of 40-year-olds found in the precarious milieus is above average. Traditional milieus can be found in all social classes (low, middle, and high) and are particularly characterized by the generational situation of the war and post-war generation. Therefore, the majority of people in these milieus are therefore aged 70 years or more (21). One possible explanation for the results found lies in the existing awareness and knowledge about noise and the place of residence. For example, Gilani and Mir (24) showed that people from noisy areas had a higher risk of exposure, and despite high literacy, such people had low awareness of noise and its health consequences, such as quality of sleep (25). Zhao et al. (26) demonstrated a connection between environmental health literacy and place of residence. Residents in a city showed a higher level of environmental health literacy than those living in rural areas. Despite these differences, they pointed out that overall, the level of environmental health literacy level was low, and awareness of environmental health risks should be increased.

Data on environmental (health) literacy is not available in Germany, but it could be deduced from the data on health literacy (27) that people from precarious milieus also have a lower level of environmental (health) literacy, and perhaps, the necessary skills to understand environmental (health) risks (28) were not sufficient. This assumption can be supported by the study by Zhao et al. (26) which also showed that education and income influence environmental health literacy. All three milieus had low social positions, with the traditional milieus being distributed across all social positions (low, medium, and high). This is why education and income were important influencing factors for these milieus. To strengthen the skills to understand environmental (health) risks, studies based on a citizen science approach are needed. For example, virtual training courses could be organized as reported by Stanifer et al. (29) or health risks in the living environment could be identified together with the residents, as done by Tuckett et al. (30). Due to the included variables this secondary data analysis could only address the environmental risk of noise. No conclusions can be drawn about other environmental risks, such as heat, which is an important factor influencing human health, especially in the context of climate change (31). Future research should also consider the consequences of the climate crisis as part of the environment.

In the 2018 Environmental Awareness Study, environmental knowledge was only analyzed in connection with the explanation of environmental awareness. It was known that an average of 5.4 of 10 questions were answered correctly. To the best of our knowledge, a more detailed analysis of milieus in relation to environmental knowledge has not yet been published (17). Based on the results of this secondary data analyses, it could be shown that precarious milieus have significantly lower environmental knowledge than traditional milieus, established milieus, the bourgeois mainstream, critical-creative milieus, and young idealists (all *p* < 0.001). Thus, significant differences were found in five out of seven of the milieus compared. No significant differences found between only precarious milieus and young pragmatists and between precarious milieus and the young detached.

With regard to the different milieus and social situation, it is difficult to make a statement in this regard as significant differences were found in both milieus with a high social situation (e.g., established milieus) and those with a middle social situation (e.g., the bourgeois mainstream). Furthermore, it should not be forgotten that people of all milieus generally answered the questions on environmental knowledge incorrectly very often. Since environmental knowledge and general knowledge are closely related (15), the results of the secondary data analysis indicate that environmental knowledge is not yet sufficiently anchored in the general knowledge of the German population. This knowledge gap could cause increased environment-related risks to health, as knowledge and experience of the social situation influence individual health behavior, increasing vulnerability to environmental risks (10). Further research is needed to investigate the differences in environmental knowledge in relation to social situation.

The multiple regression analysis showed that environment cognition (*p* = 0.008) and male gender (*p* < 0.001) mainly had a significantly influence on environmental knowledge. This result is surprising because the 2018 Environmental Awareness Study showed that in general, women and younger people in particular are more environmentally aware, so it was expected that women and younger people would also have more environmental knowledge. There seem to be differences here within the milieus. Based on other studies, it was also expected that income and education would have a significant influence on environmental knowledge. However, individual differences must also be assumed here (32).

In order to increase the environmental knowledge of the German population, the extent to which existing environmental information is generally accessible and understandable for specific target groups should be investigated in the future. This is important because understandable information is closely linked to health literacy (33) and allows health-promoting decisions to be made (27). This can be achieved through the use of easy language and infographics. Studies related to environmental health have shown that infographics are effective. For example, Ginzburg et al. (34) used an infographic to communicate traffic-related ultrafine particles in air pollution with health risks. The infographic was evaluated using a survey, focus groups, and interviews. The participants perceived the infographic as positive due to the images used and the clear formulation of the purpose. In particular, existing (communication) structures should be critically examined with regard to the needs and requirements of people from precarious milieus. For future research, we therefore suggest that content analyses of environment-related (health) information materials examine the content and the way it is communicated. However, approaches should not only be pursued to strengthen individual health literacy, but also the organizational health literacy of health professionals (35), for example, who can act as multipliers. Healthy living environments are also needed, which could be achieved through “Health in All Policies” (36).

Significant differences were found in all three subcomponents of environmental awareness. Precarious milieus had higher environmental affect than established milieus but lower affect than the critical-creative milieus and the young idealists (all *p* < 0.001). As mentioned, the precarious milieus and the established milieus differ in terms of social situation and are similar in terms of socio-historical factors (21). This means that precarious milieus are more emotionally affected by environmental issues, which is consistent with the results of the environmental health burdens in this secondary data analysis. According to the 2018 Environmental Awareness Study (17), established milieus also had the lowest environmental affect. Higher environmental effect among critical-creative milieus and the young idealists were not surprising when considering that the critical-creative milieus have a high level of interest in social issues and question them critically (21). For young idealists, sustainability is an important part of their self-image and can be linked to engagement (21). In addition, both milieus have the highest values for environmental affect (17). A study by Hajek and Koenig (37) on climate anxiety in Germany shows that it is higher among younger people. A high level of climate anxiety also goes hand in hand with a high level of fear and depression, as well as greater awareness of the effects of climate change (38). In view of the precarious milieus’ high perception of their own vulnerability to environmental health burden, future research should also analyze emotional environmental issues such as climate anxiety based on specific target groups.

The multiple regression analysis showed that environmental cognition (*p* < 0.001) and environmental behavior (*p* < 0.001) were associated with environmental affect within the precarious milieus. These results are consistent with the results of the 2018 Environmental Awareness Study (17), which showed a correlation between environmental affect, environmental cognition, and environmental behavior in the entire sample. It appears that this correlation can be found both overall and in the specific consideration of precarious milieus. The strength of this correlation could be investigated further by analyzing the other milieus.

Significant differences in environmental cognition were found between precarious milieus and the established milieus, young pragmatists, critical-creative milieus, and young idealists. Precarious milieus had significantly higher environmental cognition than established milieus and young pragmatists but lower than critical-creative milieus and young idealists (all *p* < 0.001). Despite higher social status, which is based on education and income, among other things, established milieus exhibit lower environmental cognition than precarious milieus, which is consistent with the 2018 Environmental Awareness Study (17), where established milieus had the lowest environmental cognition. In contrast, critical-creative milieus and young idealists had the highest environment cognition. The significant difference between precarious milieus and young pragmatists was surprising. Similar to young idealists, young pragmatists have medium to high social status. Young pragmatists, however, place importance in professional success and a good standard of living, which is linked to economic growth (21). This could also explain the observed difference, as those in precarious milieus feel severely restricted in their participation in both consumption and social life.

The multiple regression analysis showed that environmental affect (*p* < 0.001), environmental knowledge (*p* = 0.008), and gender (*p* = 0.005) were associated with environmental cognition within the precarious milieus. Only the connection with environmental affect was in line with the 2018 Environmental Awareness Study (17). The influence of male gender within the precarious milieus is surprising as the 2018 Environmental Awareness Study (17) showed a higher level of environmental awareness among women. The connection between environmental cognition and environmental knowledge is not surprising when considering that environmental cognition reflects attitudes toward environmental issues based on the judgment of factual statements.

The finding that people from precarious milieus had less environmental behavior than other milieus is consistent with the general results of the 2018 Environmental Awareness Study (17). The present secondary data analysis showed that precarious milieus had significantly less environmental behavior than traditional milieus, critical-creative milieus, and young idealists (all *p* < 0.001). Notably, critical-creative milieus and young idealists had higher social status than precarious milieus. Based on the low-cost hypothesis (39), people from precarious milieus may act less environmentally friendly because the costs (time and money) to adopting environmentally friendly behavior are too high. Perhaps, food from controlled cultivation (organic farming) are perceived as too expensive, or the meaning of the eco-labels may be unknown, which might lead people in precarious milieus to use better-known products. This assumption is supported by a study by Vos et al. (40) who identified high prices and lack of skills as barriers to buying sustainable and healthy food among parents with a low socioeconomic status.

In contrast to the general results of the 2018 Environmental Awareness Study (17), no influence of female gender, age, and income on environmental behavior was found in this secondary data analysis. Instead, environmental affect (*p* < 0.001) was associated with environmental behavior, which is partly consistent with the correlations mentioned thus far. Within the precarious milieus, no connection could be found between environmental behavior and environmental cognition. The reason for this is that this study analyzed the influencing factors within the precarious milieus and not the population as a whole.

Based on the literature, a connection was expected between environmental behavior and environmental knowledge. For example, Liu et al. (41) found a positive influence of environmental knowledge on environmental attitudes, which in turn had a positive influence on environmental behavioral intention and ultimately on environmental behavior. Thus, they were able to demonstrate an indirect influence of environmental knowledge on environmental behavior. A positive correlation could not be found between environmental knowledge and environmental behavior in the present secondary data analysis. This difference can be explained by the use of different measurement methods (other questionnaires) and the target group-specific analysis of the precarious milieus in this study. This suggests that differentiated investigations must be carried out to examine the influence of environmental knowledge on environmental behavior in more detail, and the analysis could also include differences between different population groups, such as precarious milieus. In particular, life satisfaction (42) and the influence of physical and social living environments (42) should be considered in future studies of precarious milieus as they are more frequently affected by environmental health risks (9).

Environmental affect and environmental cognition play a particularly important role in the precarious milieus as they influenced two of the four factors investigated. Environmental affect is associated with environmental cognition and environmental behavior, while environmental cognition is associated with environmental knowledge and environmental affect. To be able to classify the findings, it is important to consider environmental awareness as a whole, which includes a combination of environmental affect (emotional involvement), environmental cognition (environmental attitudes), and environmental behavior (active action) (13) and either excludes or includes environmental knowledge (14).

The 2018 Environmental Awareness Study and the present secondary data analysis show that socio-demographic factors such as gender can also be influencing factors. The multiple regression analysis of environmental knowledge and the three sub-components of environmental awareness illustrates how milieus should be viewed individually. It can be seen that the influencing factors have different significance at the milieu level than in the overall analysis, as was the case in the 2018 Environmental Awareness Study. It is particularly conspicuous that the present secondary data analysis often found a significant difference between precarious milieus and established milieus, critical-creative milieus, and young idealists.

Differences can be seen above all in the comparison of low social situation and medium to high social situation. Due to different life situations, it is essential to consider specific target groups when looking at the perception of environmental health burdens, environmental knowledge, and environmental awareness. Therefore, future research should use a lifeworld-oriented approach (43) to investigate the various factors within precarious milieus regarding environmental awareness and environmental knowledge in more detail. Such research could help to reduce environmental health inequalities and contribute to environmental justice.

### 4.1 Limitations

Secondary data analyses offer a good starting point to build on existing studies, but there are also some limitations. One challenge is that the data collection was not developed to answer the research questions in this study, which means some necessary information was missing in the data or was not ideally operationalized or available. For example, individual opportunities to implement environmental behavior were not taken into account.

In addition, it was not possible to influence the knowledge questions that were used to determine environmental knowledge. Because the 2018 Environmental Awareness Study did not focus on environmental knowledge, other important information was also missing, such as the availability of information (content and mode of delivery), which could have helped to examine environmental knowledge in a more differentiated way. The division into milieus was also predetermined, which made differentiated consideration within the milieus difficult. It was not possible to design subgroups, which is why precarious milieus had to be regarded as a homogeneous group, although differences within the milieu can be assumed. The different sizes of the eight milieus may also represent a limitation.

Another limitation is that the environmental awareness scale has been further developed in the meantime. Analyses have shown that the items on environmental affect and environmental cognition correlate almost perfectly, which is why the authors of the Environmental Awareness Study assume a two-dimensional model composed of an affective-cognitive component and a conative component (44). In the 2018 Environmental Awareness Study and in this secondary data analysis, environmental awareness was understood as a three-dimensional model. Further research is needed to develop a comprehensive scale of environmental awareness that goes beyond the assumed dimensions of environmental affect, environmental cognition and environmental behavior.

As the results relate to Germany, it must be questioned whether the results are also transferable to other countries. In particular, the classification according to milieus could also be different in other countries.

Nevertheless, the aspect of environmental justice should be considered as a larger context for our results. As this was a German study, it should be kept in mind that the discussion on environmental justice differs from that in the USA because the discussion there is much broader. This does not mean that there are no similarities because social situation and one’s own vulnerability also play important roles in the significance of environmental burden, not only the local living environment in the form of environmental (health) risks and environmental resources (10).

## 5 Conclusion

The differences in the perception of environmental health burdens, environmental knowledge, and environmental awareness indicated that group-specific education and support structures are required. Research on environmental health literacy in Germany can provide initial clues to better understand the awareness and perception of environmental health burdens. In particular, there still seems to be insufficient environmental knowledge among the German population, and further research is needed to find out the reasons for this. Therefore, it should be a political priority to increase knowledge among the whole population through group-specific offers and promotion.

One way to achieve this could be through adapted environmental communication aimed at different milieus. In this context, the use of easy language and visual communication for people from precarious milieus would be conceivable when taking into account the diversity of these milieus. It is also important to investigate other factors within precarious milieus that could influence environmental awareness and environmental knowledge. Future research should also look at how environmental communication can be successful for specific groups by identifying needs and adapting communication strategies to minimize environmental injustice in Germany.

## Data Availability

The raw data are freely accessible via the platform of “GESIS - Leibniz Institute for the Social Sciences” at https://search.gesis.org/research_data/ZA7493.
